# Supplementary figures and images for: The presence of enteropathy in HIV infected children on antiretroviral therapy in Malawi
Source: PLoS One. 2024 Feb 8;19(2):e0298310. doi: 10.1371/journal.pone.0298310 (PMC10852317; doi:10.1371/journal.pone.0298310)

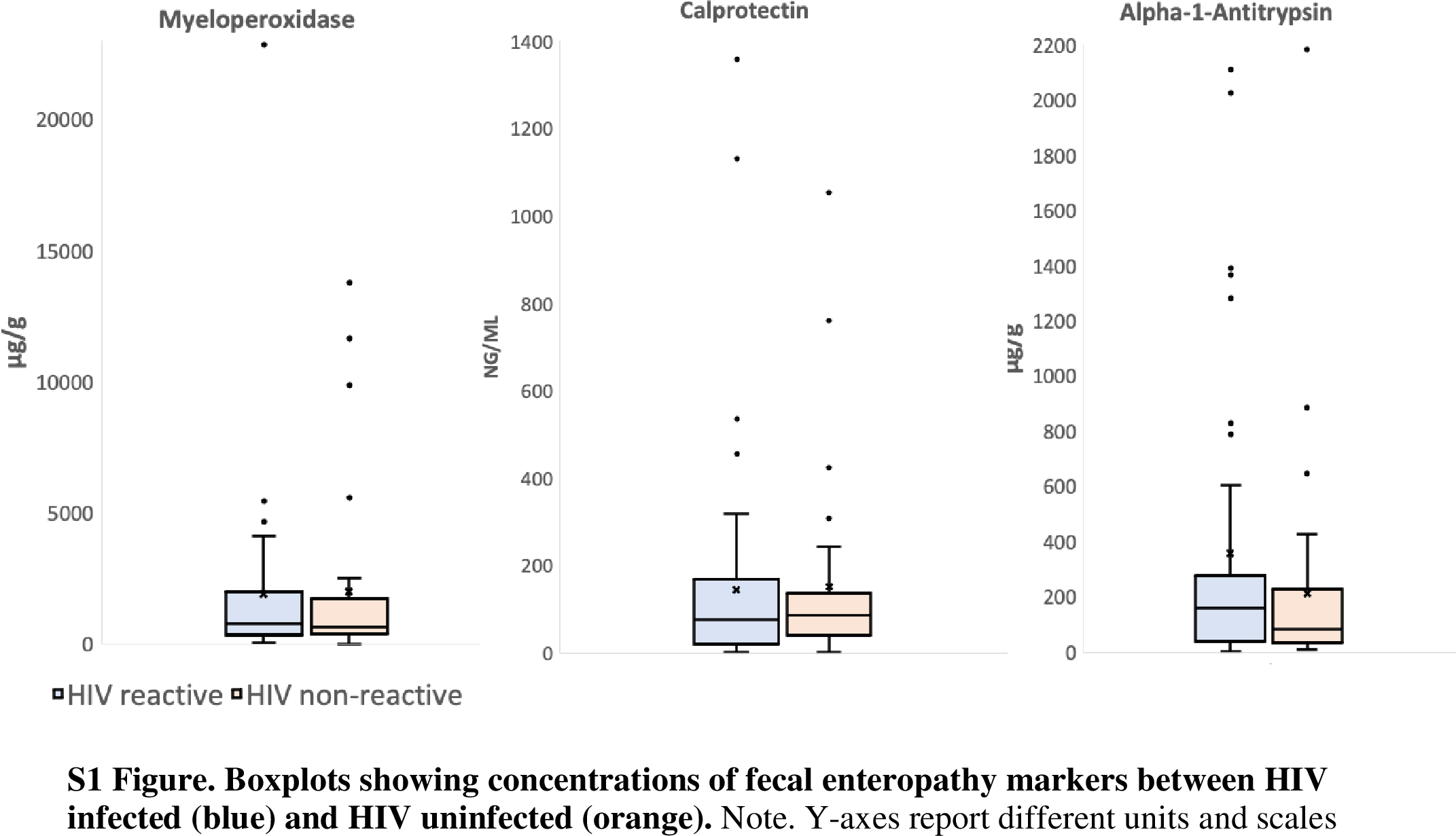

Supplement: S1 Fig — Note. Y-axes report different units and scales. (TIF) [file pone.0298310.s001.tif]

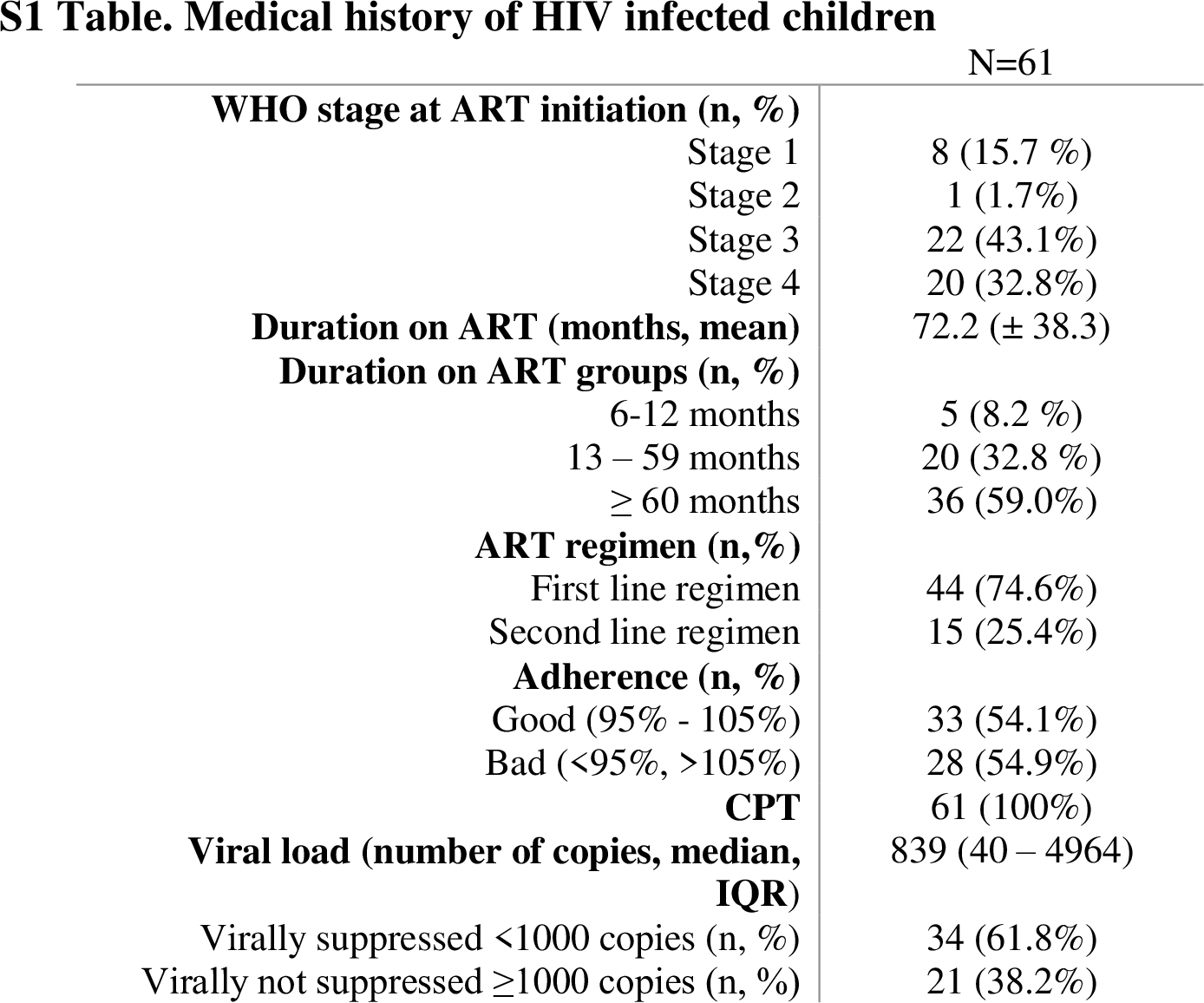

Supplement: S1 Table — (TIF) [file pone.0298310.s002.tif]

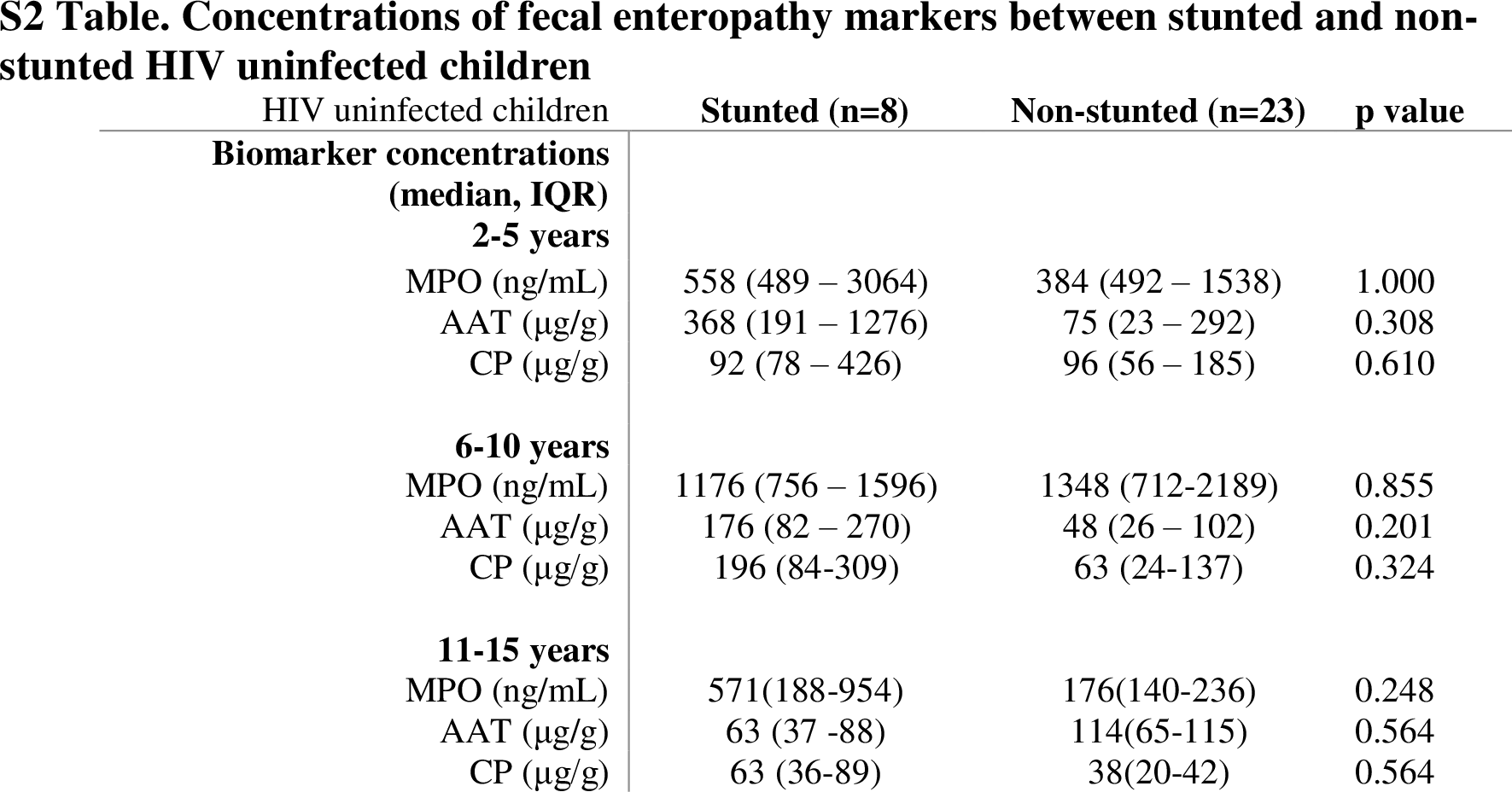

Supplement: S2 Table — (TIF) [file pone.0298310.s003.tif]

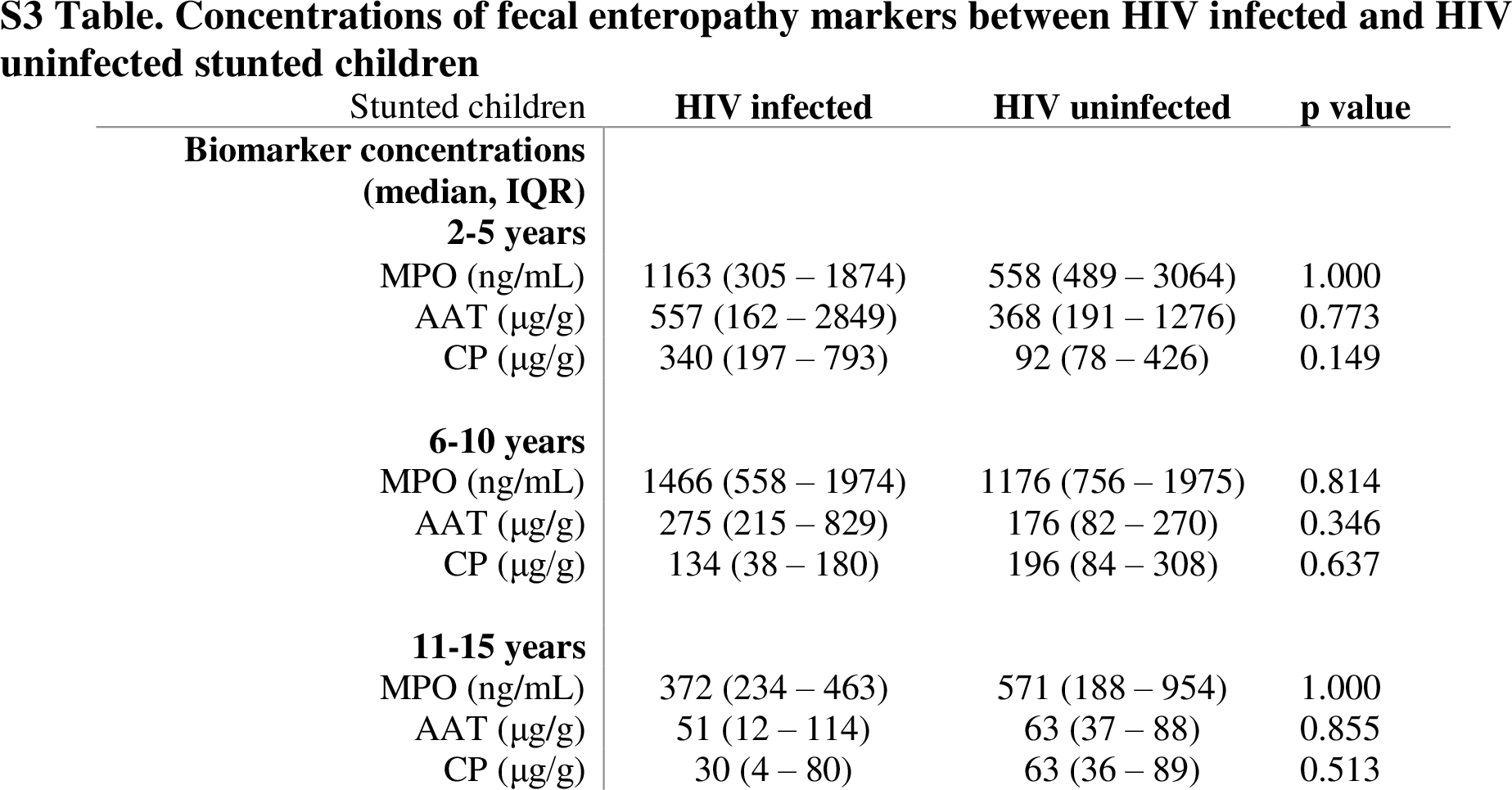

Supplement: S3 Table — (TIF) [file pone.0298310.s004.tif]
