# Supplementary material for: The presence of enteropathy in HIV infected children on antiretroviral therapy in Malawi
Source: PLoS One. 2024 Feb 8;19(2):e0298310. doi: 10.1371/journal.pone.0298310 (PMC10852317; doi:10.1371/journal.pone.0298310)
Supplement: Attachment — Submitted filename: Response to reviewers.pdf [file pone.0298310.s005.pdf]

Editorial Office PLOS
Nine Hills Road
Cambridge, CB2 1GE
United Kingdom

Subject: Manuscript Revision - The presence of enteropathy in HIV infected children on antiretroviral therapy in Malawi.

Amsterdam, 27-11-2023

Dear Editor, dear dr Furqan Kabir,

I would like to express my gratitude to you and the esteemed reviewers for the constructive feedback provided on my manuscript entitled " The presence of enteropathy in HIV infected children on antiretroviral therapy in Malawi " I appreciate the time and effort invested in the review process. I have carefully considered each of the reviewers' comments and suggestions and would like to address them in this rebuttal letter.

Reviewer #1:

- *Comment:* reviewer #1 asked questions about why we used these biomarkers when Neopterin, LR of LM ratios are also counted as very promising biomarkers of EED?

  Answer: Due to logistical and financial constraints, we were restricted to assessing only three biomarkers in our study. Neopterin, together with the two markers we used, CP and MPO, are indicative of intestinal inflammation. The decision to opt for CP and MPO over NEO was based on the comparable nature of their results and growth outcome evaluations. Given that we had already employed two biomarkers focusing on intestinal inflammation, we included AAT, assessing intestinal permeability, to present a more comprehensive perspective on intestinal functionality. While lactulose-mannitol (L:M) and lactulose-rhamnose (L:R) ratios are also reflective of permeability, their utilization is hampered by numerous limitations stemming from inconsistent associations with growth outcomes and the variability in L:M test results across different laboratories and platforms[1,2].
  1. Denno et al. Clinical Infectious Diseases 2014;59:S213–9.
  2. Harper et al.Journal of XYZ 2018.

- *Comment:* Why did the sample collection stop at a number of 114 children? Please justify. Was any sample size calculated for this or it was just an arbitrary decision?

- *Answer:* Based on a power calculation, we aimed to recruit a total of 30 participants for each arm to detect a difference in primary outcome with $\alpha = 0.05$ and 80% power, resulting in a total of 90 participants. Recruitment went very smoothly and faster than

expected. Anthropometry and clinical data were collected from 253 children. However, because collecting stool samples proved to be more difficult logistically, we were only able to collect a stool sample of 114.

- *Comment:* A brief introduction of EED with its histological features would be good.
- *Answer:* We have amended the introduction and added a few lines regarding the pathophysiology of EED to the manuscript. (*please see page 3 line 67-73 of the revised manuscript)*

- *Comment:* "This study is a continuation of a previous cross-sectional pilot study"- please add few lines regarding that pilot study for the readers to understand the context well.

  Answer: An explanation of this is admitted in the manuscript. (*please see page 4 line 97-100 of the revised manuscript)*

Reviewer #2:

- *Comment:* Abstract: Line 34: The author needs to mention the rationale of this study. + Line 78: The author needs to mention the rationale of this study. The need to state the specific knowledge gap in this field.

  Answer: We have revised the abstract and introduction section to properly reflect the study's rationale. (*please see page 2 line 34-39 and page 4 line 84-92 of the revised manuscript)*

- *Comment:* Methods:
  Line 95: Is the questionnaire validated?

  Answer: The questions in the 'questionnaire' are not validated but are very normal medical questions regarding housing, people in the family and questions regarding initiation of and compliance with ART's. To prevent misunderstandings, we have removed the word or reference to a 'questionnaire' but framed it as 'We collected both social and medical histories from all participants by using standard medical questions'. (*please see page 5 line 106-107 of the revised manuscript)*

- *Comment:* Need to declare the data sharing policy at the end of manuscript.

Answer: We recognize the importance of transparency and open access in scientific research and we are committed to prioritize the privacy and confidentiality of sensitive information. We believe this is the case in our manuscript given that the data includes details about the HIV status of vulnerable children and their families. When making this potentially stigmatizing and sensitive data/information publicly available, this can compromise the privacy and well-being of this vulnerable group. We would very much prefer not to make the data publicly available but upon request. We have added a non-author point of contact where data requests may be made. We very much hope this is possible and are looking forward to your response.

We added to following statement to the manuscript (page 15): "Data sharing requests can be directed to dr W.P. Voskuijl at w.p.voskuijl@amsterdamumc.nl or to the secretariat of The Amsterdam Institute for Global Health and Development at secretariat@aighd.org. Requests will be evaluated based on their scientific merit, the integrity of the research purpose, and the protection of participant confidentiality. Access to the data will be provided in compliance with applicable privacy and ethical standards."

Thank you once again for the opportunity to publish in PLOS ONE. I look forward to your final decision and appreciate your consideration of the revised manuscript.

Sincerely,

Dr. Julia Blaauw (MD) and dr Wieger Voskuijl (MD, PhD)
Amsterdam Institute for Global Health and Development, Department of Global Child Health, Amsterdam University Medical Centers
